# The protozoan parasite *Theileria annulata* alters the differentiation state of the infected macrophage and suppresses musculoaponeurotic fibrosarcoma oncogene (MAF) transcription factors

**DOI:** 10.1016/j.ijpara.2009.02.020

**Published:** 2009-08

**Authors:** Kirsty Jensen, Giles D. Makins, Anna Kaliszewska, Martin J. Hulme, Edith Paxton, Elizabeth J. Glass

**Affiliations:** Division of Genetics & Genomics, The Roslin Institute and Royal (Dick) School of Veterinary Studies, University of Edinburgh, Roslin Biocentre, Midlothian EH25 9PS, UK

**Keywords:** *Theileria annulata*, Bovine, Host-pathogen interactions, MAF transcription factors, Macrophage differentiation

## Abstract

The tick-borne protozoan parasite *Theileria annulata* causes a debilitating disease of cattle called Tropical Theileriosis. The parasite predominantly invades bovine macrophages (mϕ) and induces host cell transformation by a mechanism that has not been fully elucidated. Infection is associated with loss of characteristic mϕ functions and phenotypic markers, indicative of host cell de-differentiation. We have investigated the effect of *T. annulata* infection on the expression of the mϕ differentiation marker c-maf. The up-regulation of c-maf mRNA levels observed during bovine monocyte differentiation to mϕ was suppressed by *T. annulata* infection. Furthermore, mRNA levels for c-maf and the closely related transcription factor mafB were significantly lower in established *T. annulata*-infected cell-lines than in bovine monocyte-derived mϕ. Treatment of *T. annulata*-infected cells with the theileriacidal drug buparvaquone induced up-regulation of c-maf and mafB, which correlated with altered expression of down-stream target genes, e.g. up-regulation of integrin B7 and down-regulation of IL12A. Furthermore, *T. annulata* infection is associated with the suppression of the transcription factors, Pu.1 and RUNX1, and colony stimulating factor 1 receptor (CSF1R) which are also involved in the regulation of monocyte/mϕ differentiation. We believe these results provide the first direct evidence that *T. annulata* modulates the host mϕ differentiation state, which may diminish the defence capabilities of the infected cell and/or promote cell proliferation. Musculoaponeurotic fibrosarcoma oncogene (MAF) transcription factors play an important role in cell proliferation, differentiation and survival; therefore, regulation of these genes may be a major mechanism employed by *T. annulata* to survive within the infected mϕ.

## 1 Introduction

The tick-borne apicomplexan parasite *Theileria annulata* is the causative agent of the cattle disease Tropical Theileriosis, which is of major economic importance in countries in Northern Africa and Asia. The infectious sporozoite stage of the parasite exhibits cell tropism, predominantly invading bovine macrophages (mϕ) and to a lesser extent B lymphocytes (Glass et al., 1989). Within these cells the parasite differentiates into the multinucleated schizont stage, which is associated with much of the disease pathology.

A unique feature of *T. annulata,* and the closely related parasite *Theileria parva*, is the ability of the schizont stage to induce host cell transformation without involving the integration of parasite DNA into the host genome. This leads to continuous proliferation of the infected cell and parasite (Spooner et al., 1989). These cells disrupt the morphology and function of the lymph node draining the site of infection (Campbell et al., 1995). In addition, infected cells disseminate through lymphoid tissue and non-lymphoid organs including the heart, lungs and brain, causing haemorrhagic lesions (Forsyth et al., 1999). The mechanism behind the transformation event has been extensively studied, but is not fully understood (reviewed Dobbelaere and Kuenzi, 2004; Heussler et al., 2006; Plattner and Soldati-Favre, 2008). This work has revealed an extremely complex network of interactions between the parasite and host cell, involving many cellular pathways, including; NF-κB (Heussler et al., 2002), c-myc (Dessauge et al., 2005), SRC-related tyrosine kinase (Eichhorn and Dobbelaere, 1994), c-jun NH2-terminal kinase (JNK) (Galley et al., 1997; Chaussepied et al., 1998), phosphoinositide-3 kinase (Baumgartner et al., 2000), protein kinase A (Guergnon et al., 2006) and Notch (Chaussepied et al., 2006).

*Theileria annulata* infection alters the phenotype and function of the host mϕ (reviewed by Glass and Jensen, 2007). Phenotypic changes include the down-regulation of mϕ markers CD14 and CD11b, together with increased surface expression of CD2 and bovine major histocompatibility complex (BoLA) class II genes (Glass and Spooner, 1990; Brown et al., 1995; Sager et al., 1997; Glass and Jensen, 2007). Several mϕ functions are impaired by *T. annulata*, including phagocytosis and the production of nitric oxide, superoxide and pro-inflammatory cytokines in response to stimulation by phorbol 12-myristate 13-acetate and lipopolysaccharide (LPS) (Sager et al., 1997). Transformation of the infected mϕ is reversible and requires the presence of live parasites (Sager et al., 1997). Treatment of infected cells with the theileriacidal drug buparvaquone results in a decrease in cell-cycling, increased surface expression of CD14 and CD11b, and the reacquisition of phagocytosis function (Sager et al., 1997).

Mϕ are an end product of the differentiation of myeloid progenitor cells via blood monocytes. Monocytes have the potential to differentiate into different subsets of mϕ and dendritic cells (DC), which play a multitude of important roles in the immune response (Iwasaki and Akashi, 2007; Geissmann et al., 2008; Serbina et al., 2008). It has been postulated, from the phenotypic and functional changes associated with *T. annulata* infection, that the parasite induces the mϕ to revert back to a de-differentiated state, which may be a strategy utilized by the parasite to subvert the mϕ defence response (Sager et al., 1997). However, *T. annulata-*infected cells exhibit up-regulation of BoLA class II molecules and pro-inflammatory cytokines (Brown et al., 1995; McGuire et al., 2004), enhanced antigen-presenting capabilities (Glass and Spooner, 1990) and induce neighboring T lymphocytes, including naïve T cells, to activate and proliferate (Campbell et al., 1995). Therefore, the infected mϕ does not resemble monocytes or myeloid progenitor cells.

The differentiation of mϕ from myeloid progenitor cells is a tightly controlled process regulated by transcription factor activity (reviewed by Hume and Himes, 2003; Friedman, 2007). Transcription factors involved include the Ets family member spleen focus forming virus (SFFV) proviral integration oncogene spi1 (Pu.1), the core binding family member runt-related transcription factor 1 (RUNX1) and two members of the musculoaponeurotic fibrosarcoma oncogene (MAF) transcription factor family, c-MAF and MAFB. Expression of human c-MAF increases progressively from myeloid progenitor cells to mϕ and is, therefore, a good marker of human mϕ differentiation (Martinez et al., 2006; Liu et al., 2008). Transcriptome profiling of bovine monocytes following infection with *T. annulata* revealed that c-MAF was one of the most differentially regulated genes (Jensen et al., 2008). In addition to its role in differentiation, c-MAF was originally identified as an oncogene (Kataoka et al., 1993) and therefore, may play a role in the transformation of the *T. annulata-*infected cell. Therefore, we have further investigated the effect of *T. annulata* infection on the expression of c-MAF and other transcription factors in bovine monocytes and mϕ. The study has revealed that the expression of both MAF transcription factors and other transcription factors involved in the regulation of monocyte/mϕ differentiation are suppressed by the presence of *T. annulata*. This supports the hypothesis that the parasite alters the differentiation state of the host cell, which may be essential for parasite-induced survival and proliferation.

## 2 Materials and methods

### 2.1 Animals

Peripheral monocytes were isolated from four Sahiwal cattle (*Bos indicus*) maintained off pasture at the Centre for Tropical Veterinary Medicine (CTVM), University of Edinburgh, UK, which have been described previously (Jensen et al., 2006). Cells were also collected from 12 female Holstein–Friesian cattle (*Bos taurus*) maintained at The Roslin Institute, University of Edinburgh, UK. These Holstein–Friesian cattle were between 3 and 5 years of age and kept on pasture. All experimental protocols were authorized under the UK Animals (Scientific Procedures) Act, 1986.

### 2.2 Isolation of bovine monocytes

Peripheral blood was collected aseptically into acid citrate dextrose (ACD) and immediately stored on ice. Peripheral blood mononuclear cells (PBMC) were separated under cold conditions by density gradient centrifugation as described previously (Jensen et al., 2006). Peripheral monocytes were isolated from PBMC by positive selection using the monoclonal antibody IL-A24, which recognizes signal-regulatory protein alpha (SIRPA) (Brooke et al., 1998), and the MACS system (Miltenyi Biotec) as described previously (Jensen et al., 2006). Fluorescence-activated cell sorting (FACS) analysis confirmed that the cell purity exceeded 95% (data not shown).

### 2.3 Infection and stimulation of bovine monocytes

Resting peripheral monocytes from five cattle were resuspended at 4 × 10^6^ cells/ml in RPMI-1640 medium supplemented with 20% FBS and aliquoted into a 12-well plate. One half of each sample were infected with *T. annulata* (Ankara) sporozoites in homogenized infected tick (*Hyalomma anatolicum*) preparations (kindly provided by Dr. Patricia Preston, University of Edinburgh, UK), as described previously (Jensen et al., 2008). Briefly, an equal volume of sporozoite suspension at 0.25 tick equivalents/ml in RPMI-1640 medium supplemented with 40% FBS was added to the monocytes and incubated at 37 °C in a 5% CO_2_ incubator. The sporozoite concentration had been previously optimized to maximize the percentage of monocytes being infected (data not shown). As a medium-only negative control, an equal volume of RPMI-1640 medium supplemented with 40% FBS was added to the other half of each monocyte sample. Cells were harvested at 0 and 72 h p.i. The infected monocytes were pelleted, washed with PBS and RNA was immediately isolated from the cells.

In a separate experiment, monocytes from four cattle were purified as described above and stimulated with homogenized uninfected *H. anatolicum* (kindly provided by Dr. Alan Walker, University of Edinburgh, UK), prepared using a similar protocol to that used to generate infected tick preparations (Brown, 1987). Cells were harvested at 0 and 72 h post stimulation and RNA was immediately isolated from the cells.

### 2.4 Preparation of bovine monocyte-derived macrophages

Bovine mϕ were generated from the peripheral blood of eight Holstein–Friesian cattle as described previously (Jungi et al., 1996). Briefly, blood was collected aseptically into ACD and buffy coats were separated by centrifugation. The resulting cells were washed with citrate buffer (30 mM citric acid, 0.6% NaCl, 3 mM KCl, 4.3 mM Glucose) to remove fibrinogen, followed by hypotonic lysis of erythrocytes. PBMC were separated by density gradient centrifugation on Lymphoprep (Axis-Shield) and resuspended at 4–5 × 10^6^ cells/ml in Iscove’s modified Dulbecco’s medium (IMDM) (Invitrogen) supplemented with GlutaMax™ (Invitrogen), 25 mM Hepes, 100 IU/ml penicillin, 100 μg/ml streptomycin, 10 mM sodium pyruvate, 1% minimum essential medium (MEM) vitamins (Invitrogen), 1% non-essential amino acids (Invitrogen), 50 μM β-mercaptoethanol and 20% FBS. The purified PBMC were cultured in non-adherent Teflon bags for 7 days at 37 °C in a 5% CO_2_ incubator, during which time the monocytes differentiated into mϕ (Jungi et al., 1996). Cells were resuspended in fresh medium supplemented as above, except that the FBS concentration was reduced to 2%. Mϕ were purified by selective adherence overnight to 6-well plates.

### 2.5 Cell lines

Two sets of *T. annulata*-infected cell-lines were used in this study. The first set comprised 10 previously established *T. annulata* (Hisar) infected cell-lines, between passages 4 and 7, which were established ex vivo from the peripheral blood of Sahiwal and Holstein–Friesian calves following experimental infection (McGuire et al., 2004). The second set comprised five *T. annulata*-infected cell-lines generated from in vitro infection of purified Holstein–Friesian or Sahiwal peripheral monocytes, prepared as described above. The cells were infected with the Ankara, Hisar or Gharb strains of *T. annulata*. All cell-line cultures, between passages 4 and 8, were maintained in RPMI-1640 medium supplemented with 10% FBS, 2 mM l-glutamine and 50 μM β-mercaptoethanol and cultured at 37 °C in a 5% CO_2_ incubator.

The parasites were eliminated from cells by treatment with the theileriacidal drug buparvaquone. The cells, at 1 × 10^6^ cells/ml, were treated with 25 ng/ml buparvaquone (kindly supplied by Dr. MacHugh, CTVM) diluted in 10 mM potassium hydroxide (KOH) in 100% ethanol for 72 h. In addition negative control samples, treated with an equal volume 10 mM KOH in 100% ethanol, were prepared.

### 2.6 Total RNA extraction

Total RNA was extracted from all monocyte, bovine mϕ and *T. annulata*-infected cell-line samples using the RNeasy mini kit (Qiagen) according to the manufacturer’s instructions. The resulting RNA was treated with DNA-free (Ambion) according to the manufacturer’s instructions to remove any contaminating genomic DNA. The quality and quantity of the resulting RNA was determined by gel electrophoresis and using a Nanodrop spectrophotometer. First strand cDNA was reverse transcribed from 0.5 μg total RNA using oligo (dT) primer and Superscript II (Invitrogen) according to the manufacturer’s instructions. The resulting cDNA was diluted 1:25 for quantitative reverse transcription-PCR (qRT-PCR) analysis.

### 2.7 Quantitative RT-PCR

Oligonucleotides were designed for each gene using Primer3 (Rozen and Skaletsky, 2000) and Netprimer (Biosoft International) software (Table 1). The mRNA levels of each transcript were quantified by qPCR using the Platinum SYBR Green qPCR Supermix UDG kit (Invitrogen). Reactions were carried out in 20 μl vols. containing; 1× supermix (SYBR Green, Platinum taq DNA polymerase, dNTPs, uracil DNA glycosylase (UDG) and stabilizers), 0.4 μl Rox dye, 1 μl forward and reverse primers at predetermined optimal concentrations and 5 μl diluted cDNA. Amplification and detection of products was carried out using a Mx3000P PCR machine (Stratagene) with the following cycle profile: 50 °C for 2 min, 95 °C for 2 min followed by 40 cycles of 95 °C for 15 s and 60 °C for 30 s. The detection of a single product was verified by dissociation curve analysis. Each PCR experiment was carried out in triplicate and contained several non-template controls and a log_10_ dilution series of activated monocyte cDNA or plasmids containing the sequence of interest. The relative quantities of mRNA were calculated using the method described by Pfaffl (2001). The qRT-PCR results for zinc finger protein 828 (ZNF828), previously known as Chromosome 13 open reading frame 8, were used to calculate differences in the template RNA levels and thereby standardize the results for the genes of interest. ZNF828 was previously selected from microarray and qRT-PCR analyses as a constitutively and moderately expressed gene in activated, *T. annulata*-infected and resting Holstein–Friesian- and Sahiwal-derived monocytes (Jensen et al., 2006). The relative quantity values were transformed on the log_2_ scale before statistical analyses to stabilize the variance. The effects of cell differentiation, infection and buparvaquone treatment were examined by *t*-test analysis (Genstat 10.2, Lawes Agricultural Trust, Rothamsted).

## 3 Results

### 3.1 c-MAF is a marker of bovine macrophage differentiation

**Fig. 1:**
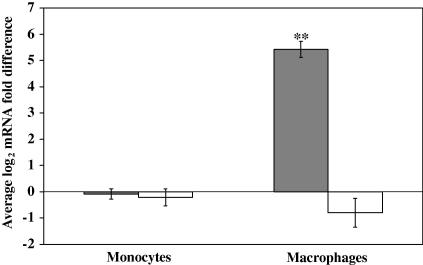
The musculoaponeurotic fibrosarcoma oncogene (MAF) transcription factor c-MAF is a marker of macrophage (mϕ) differentiation. Shown are average log_2_ mRNA fold difference in bovine monocytes and bovine monocyte-derived mϕ compared with a standard resting monocyte sample for c-MAF (grey bars) and closely related transcription factor MAFB (white bars) The error bars indicate the standard error for eight biological replicates. The statistical significance of the difference in mRNA levels between the cell types is indicated and ** denotes *P* ⩽ 0.001.

The expression of c-MAF has previously been shown to be a marker of human mϕ differentiation (Martinez et al., 2006; Liu et al., 2008). To investigate whether this was also true of bovine mϕ, the expression of c-MAF and MAFB was measured in bovine mϕ and compared with that found in freshly isolated, resting monocytes. Bovine mϕ were generated from peripheral monocytes by culturing for 7 days in Teflon bags as described previously (Jungi et al., 1996) and exhibited typical mϕ phenotypic characteristics, e.g. morphology and cell marker expression (data not shown). The expression of c-MAF and MAFB was measured by qRT-PCR and found to be consistent between bovine monocyte samples (Fig. 1). The level of c-MAF expression was up-regulated in bovine mϕ, by on average 43-fold, compared with freshly isolated monocytes (*P* < 0.001). MAFB expression in bovine mϕ did not differ significantly from that observed in resting monocytes. This result confirms that c-MAF, but not MAFB, is a marker of bovine monocyte to mϕ differentiation.

### 3.2 In vitro *T. annulata* infection suppresses the transcriptional up-regulation of c-MAF induced by monocyte differentiation

**Fig. 2:**
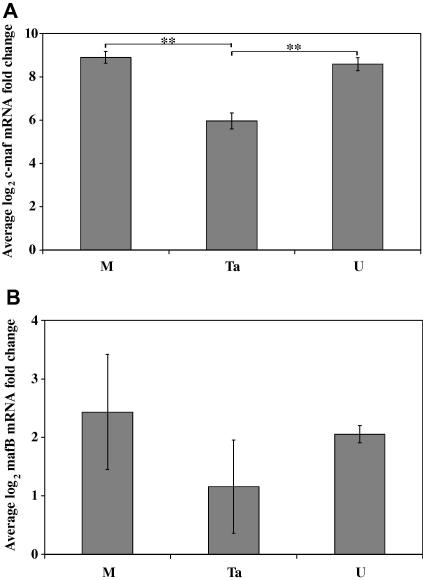
The expression of musculoaponeurotic fibrosarcoma oncogene (MAF) transcription factors c-MAF and MAFB 72 h post-activation and *Theileria annulata* infection. Quantitative reverse transcription-PCR analysis of (A) c-MAF and (B) MAFB average log_2_ mRNA fold change after 72 h in culture compared with resting monocytes. M denotes medium only, Ta denotes *T. annulata*-infected tick preparations and U denotes uninfected tick preparations. The error bars indicate the standard error for monocytes isolated from four cattle. The statistical significance of the difference in fold change is indicated and ** denotes *P* ⩽ 0.001.

The schizont stage of *T. annulata*, which is the stage associated with host cell proliferation, develops within 2 days after sporozoite infection of the mϕ (Jura et al., 1983). To investigate whether the regulation of mϕ differentiation is required for host cell transformation, c-MAF mRNA levels were measured in monocytes cultured in the presence or absence of *T. annulata* sporozoites for 3 days, before parasite-induced host cell proliferation becomes apparent. The bovine monocytes were cultured for 3 days in tissue culture plates, which induces an intermediate differentiation state in human monocytes (Martinez et al., 2006; Lehtonen et al., 2007). The expression of c-MAF was observed to increase by on average 481-fold after this period (Fig. 2A, M), which was significantly greater than observed after 7 days culture in Teflon bags (Fig. 1). This discrepancy may be due to the different culture conditions or may result from c-MAF levels decreasing in the latter stages of differentiation.

Compared with the medium-only control c-MAF levels rose by statistically significantly less (*P* < 0.001), on average 62-fold, when cultured for 3 days in the presence of *T. annulata* sporozoites (Fig. 2A, Ta). The sporozoites were prepared from infected ticks, which may contribute to the suppression of c-MAF expression. However, monocytes cultured for 3 days in medium and uninfected tick preparations exhibited similar c-MAF levels to cells cultured in medium only (Fig. 2A, U). We believe these results provide the first direct evidence that *T. annulata* infection affects the expression of the differentiation marker c-MAF, which suggests that the parasite has affected the differentiation of monocytes to mϕ induced by cells being in culture. However, due to possible down-regulation of c-MAF levels during the latter stages of mϕ differentiation, it is unclear whether *T. annulata* has inhibited or accelerated mϕ differentiation.

### 3.3 *Theileria annulata* infection does not affect MAFB expression during monocyte differentiation

The effect of *T. annulata* infection, medium and tick debris on MAFB expression was also investigated. After 3 days in culture MAFB mRNA levels were moderately up-regulated, between 3- and 9-fold by all three stimuli (Fig. 2B), similar to levels seen previously (Fig. 1). However, there was no significant difference between the responses to the three stimuli. The wide variation observed between the biological replicates results from the use of cells isolated from different animals from an out-bred population and the large scale of the graph. Therefore, c-MAF and MAFB transcription are differentially regulated during monocyte to mϕ differentiation and there is no evidence that *T. annulata* modulates the expression of MAFB during early infection.

### 3.4 c-MAF and MAFB mRNA levels are lower in T. annulata infected cell-lines than in uninfected macrophages

**Fig. 3:**
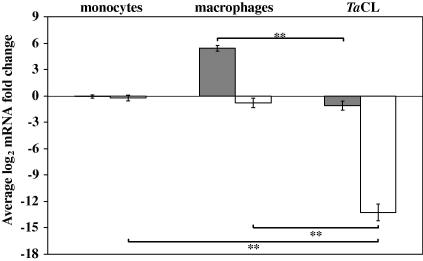
The expression of musculoaponeurotic fibrosarcoma oncogene (MAF) transcription factors MAFB and c-MAF is suppressed in *Theileria annulata*-infected cell-lines. Average log_2_ c-MAF (grey bars) and MAFB (white bars) mRNA fold difference in bovine monocytes, bovine monocyte-derived macrophages (mϕ) and *T. annulata-*infected cell-lines compared with a standard resting monocyte sample. The error bars indicate the standard error for eight biological replicates for monocytes and bovine monocyte-derived mϕ and 10 ex vivo derived *T. annulata*-infected cell-lines. The statistical significance of the difference in mRNA levels between the *T. annulata*-infected cells and uninfected monocytes or bovine monocyte-derived mϕ is indicated and ** denotes *P* ⩽ 0.001.

The expression of c-MAF and MAFB in 10 ex vivo-derived *T. annulata*-infected cell-lines was compared with that measured in resting monocytes and mϕ by qRT-PCR (Fig. 3). Levels of c-MAF mRNA in the *T. annulata*-infected cell-lines were not statistically different from those measured in resting monocytes. However, there was a statistically significant difference in c-MAF levels measured in differentiated mϕ and *T. annulata*-infected cell-lines (*P* < 0.001). In addition, MAFB mRNA levels were statistically significantly lower (*P* < 0.001) in *T. annulata*-infected cell-lines than in resting monocytes and mature mϕ, exhibiting on average a 9900-fold decrease in expression. The *T. annulata*-infected cell-lines are believed to be of mϕ origin and, therefore, these results provide evidence that transcription of both c-MAF and MAFB is suppressed by *T. annulata* infection.

### 3.5 Elimination of *T. annulata* induces up-regulation of c-MAF and MAFB

**Fig. 4:**
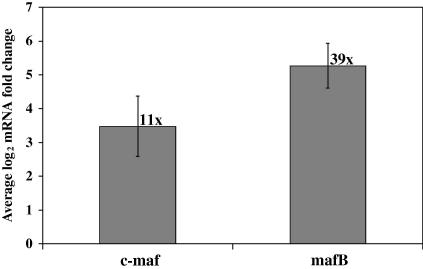
The up-regulation of musculoaponeurotic fibrosarcoma oncogene (MAF) transcription factors c-MAF and MAFB is induced by buparvaquone treatment. Shown are the log_2_ c-MAF and MAFB mRNA fold difference measured in five in vitro-derived *Theileria annulata-*infected cell-lines after 72 h treatment with 25 ng/ml buparvaquone compared with that detected in cells cultured for 72 h in an equal volume of the solution used to dilute the buparvaquone. The error bars indicate the standard error. The average fold differences are shown next to the error bars.

The previous results suggest that *T. annulata* suppresses c-MAF and MAFB expression at the transcriptional level. However, the *T. annulata*-infected cell-lines used in this study were of polyclonal origin, cultured from the blood or lymph of infected animals. Therefore, it is possible that the cell-lines were derived from cells of B lymphocyte origin, which may account for the low expression of MAF transcription factors. To investigate this, c-MAF and MAFB mRNA levels were measured in five in vitro-derived *T. annulata*-infected cell-lines of known monocyte origin. To confirm that *T. annulata* suppresses c-MAF and MAFB expression, the mRNA levels of these transcription factors were measured by qRT-PCR after culturing the cell-lines in the presence or absence of the theileriacidal drug buparvaquone. After 72 h the buparvaquone-treated cells exhibited decreased proliferation compared with the control cells (data not shown), indicative that the parasite had been killed. c-MAF mRNA levels were up-regulated in four of the *T. annulata*-infected cell-lines, by on average 11-fold (Fig. 4) compared with medium only controls. In addition, buparvaquone treatment resulted in MAFB mRNA levels being up-regulated in all five *T. annulata*-infected cell-lines by on average 39-fold (Fig. 4).

The *T. annulata*-infected cell-lines were generated from monocytes isolated from two breeds of cattle; Holstein–Friesian and Sahiwal, which are susceptible and tolerant to *T. annulata* infection, respectively (Glass et al., 2005). Although the sample size was small, there was no detectable breed difference in c-MAF and MAFB mRNA levels nor in the up-regulation of the MAF transcription factors in response to buparvaquone treatment. The cell-lines were generated from in vitro infections with three strains of *T. annulata*: Ankara, Hisar and Gharb. The up-regulation of MAFB upon buparvaquone treatment was similar in all three parasite strains. However, c-MAF was not up-regulated at 72 h post-buparvaquone treatment in the Ankara cell-line. A second Ankara strain cell-line was generated on a separate occasion from infection of purified monocytes from the same animal and buparvaquone treatment also failed to modulate c-MAF mRNA levels in this cell-line (data not shown). At this time it is not possible to determine whether the parasite strain or the animal accounts for the different c-MAF response.

To confirm that the up-regulation of c-MAF and MAFB was due to the elimination of the parasite and not an additional action of the drug buparvaquone, purified monocytes from three animals were cultured for 72 h with and without buparvaquone. There was no significant effect of buparvaquone treatment on c-MAF or MAFB mRNA levels (data not shown).

### 3.6 Buparvaquone treatment induces the transcription of genes regulated by MAF transcription factors

**Fig. 5:**
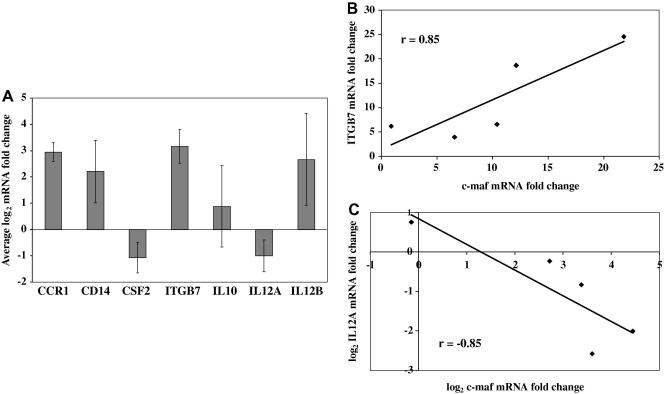
Down-stream targets of the musculoaponeurotic fibrosarcoma oncogene (MAF) transcription factors c-MAF and MAFB are up-regulated in *Theileria annulata*-infected cell-lines upon parasite elimination. (A) The average log_2_ mRNA fold change in chemokine (C–C motif) receptor 1 (CCR1), CD14, colony stimulating factor 2 (CSF2), integrin B7 (ITGB7), IL10, IL12A and IL12B levels following treatment of *T. annulata*-infected cell-lines with 25 ng/ml buparvaquone for 72 h compared with that detected in cells cultured for 72 h in an equal volume of the solution used to dilute the buparvaquone. The error bars indicate the standard error for five in vitro-derived *T. annulata*-infected cell-lines. (B) Visualization of the positive correlation between c-MAF and ITGB7 mRNA up-regulation observed in the five in vitro-derived *T. annulata*-infected cell-lines from a representative experiment. (C) Visualization of the negative correlation between c-MAF and IL12A expression changes induced by buparvaquone treatment observed in the five in vitro-derived *T. annulata*-infected cell-lines from a representative experiment. The correlation coefficient (r) is indicated.

An increase in mRNA levels does not necessarily have a biological consequence, due to potential regulation at the protein level. Unfortunately anti-bovine MAF antibodies were not available and attempts to consistently detect MAF proteins using anti-human MAF antibodies failed. Therefore, to investigate whether the increase in c-MAF and MAFB mRNA levels was associated with down-stream effects, the mRNA levels of seven genes known to be regulated by MAF transcription factors were measured by qRT-PCR. The expression of IL10 and CD14 in the five in vitro-derived *T. annulata*-infected cell-lines was not consistently altered by buparvaquone treatment (Fig. 5A) and exhibited no correlation with c-MAF or MAFB expression. However, the average mRNA levels for chemokine (C–C motif) receptor 1 and integrin B7 (ITGB7) increased 7.7- and 9.0-fold, respectively, following buparvaquone treatment (Fig. 5A). There was a statistically significant correlation between ITGB7 up-regulation and that measured for c-MAF (Fig. 5B), with a correlation coefficient of 0.85 (*P* < 0.01). In addition, there was a statistically significant correlation between CCR1 up-regulation and that measured for MAFB, with a correlation coefficient of 0.92 (*P* < 0.01) (data not shown). Furthermore, the average IL12A mRNA level decreased 2-fold following buparvaquone treatment (Fig. 5A) and there was a statistically significant correlation between IL12A down-regulation and c-MAF up-regulation, with a correlation coefficient of −0.85 (*P* < 0.01) (Fig. 5C). However, a similar correlation was not observed for IL12B, which was up- or down-regulated in different cell-lines in response to buparvaquone treatment. In addition to IL12A, there was also a significant, negative correlation between c-MAF up-regulation and the down-regulation of colony stimulating factor 2 (CSF2) following buparvaquone treatment, with a correlation coefficient of −0.83 (*P* < 0.02) (data not shown).

### 3.7 Other transcription factors regulating monocyte/macrophage differentiation and the mϕ marker CSF1R are suppressed by *T. annulata* infection

**Fig. 6:**
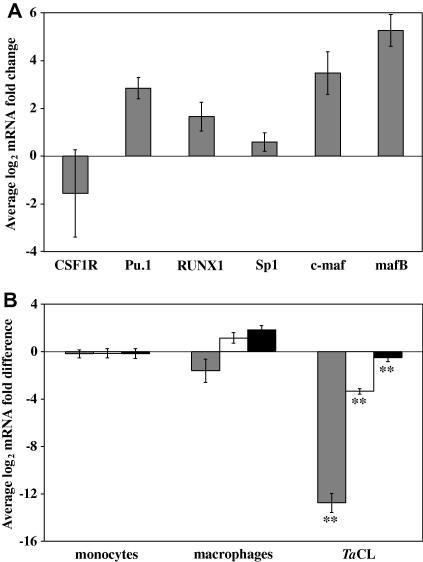
The expression of transcription factors spleen focus forming virus (SFFV) proviral integration oncogene spi1 (Pu.1) and runt-related transcription factor 1 (RUNX1) is suppressed in *Theileria annulata*-infected cell-lines. (A) Pu.1 and RUNX1 expression is up-regulated in *T. annulata*-infected cell-lines upon parasite elimination. The average log_2_ mRNA fold change in mRNA levels of colony stimulating factor 1 receptor (CSF1R), Pu.1 and RUNX1, Sp1 transcription factor (SP1), musculoaponeurotic fibrosarcoma oncogene (MAF) transcription factors c-MAF and MAFB following treatment of *Theileria annulata*-infected cell-lines with 25 ng/ml buparvaquone for 72 h compared with that detected in cells cultured for 72 h in an equal volume of the solution used to dilute the buparvaquone. The error bars indicate the standard error for five in vitro-derived *T. annulata-*infected cell-lines. (B) Average log_2_ CSF1R (grey bars), Pu.1 (white bars) and RUNX1 (black bars) mRNA fold difference in bovine monocytes, bovine monocyte-derived macrophages and *T. annulata*-infected cell-lines (*Ta*CL) compared with a standard resting monocyte sample. The error bars indicate the standard error for eight biological replicates. The statistical significance of the differences in mRNA levels between monocytes and *Ta*CL are indicated and ** denotes *P* ⩽ 0.001.

The maturation of myeloid progenitor cells to mϕ is coordinated by the activity of several transcription factors, including c-MAF and MAFB. The effect of buparvaquone treatment on the expression of three of these transcription factors; Pu.1, RUNX1 and Sp1, was measured in the five in vitro-derived *T. annulata-*infected cell-lines (Fig. 6A). Furthermore, the expression of colony stimulating factor 1 receptor (CSF1R), which plays an essential role in mϕ development and is regulated by transcription factor activity, was also investigated. There was considerable variation in the expression of CSF1R in the cell-lines following buparvaquone treatment, with either increased and reduced expression in different cell-lines. In contrast, the expression of Sp1 transcription factor was not significantly altered by buparvaquone treatment. Increased expression of Pu.1 and RUNX1 was induced by buparvaquone treatment, by on average 7.2- and 3.2-fold, respectively. This result provides further evidence that *T. annulata* modulates the differentiation state of the mϕ at the transcriptional level. However, the suppression of these transcription factors was statistically significantly less than observed for MAFB (*P* < 0.02).

To further investigate the involvement of *T. annulata* in the regulation of Pu.1 and RUNX1 mRNA levels their expression, together with that of CSF1R, was compared in bovine monocytes, mϕ and *T. annulata-*infected cell-lines (Fig. 6B). RUNX1 mRNA levels were statistically significantly higher in mϕ compared with monocytes, with on average 3.6-fold higher expression (*P* < 0.05). RUNX1 expression in *T. annulata-*infected cells was not significantly different from that measured in monocytes, but was statistically significantly lower than the mRNA level detected in mϕ (*P* < 0.001). There was no significant difference in CSF1R and Pu.1 mRNA levels in monocytes or mϕ (Fig. 6B) and CSF1R expression was very variable between mϕ samples. However, both were expressed at significantly lower levels in *T. annulata*-infected cells compared to monocytes and mϕ. Pu.1 mRNA levels were 10-fold lower in *T. annulata* infected cells than monocytes (*P* < 0.001), whilst CSF1R expression was over 6900-fold lower than that measured in monocytes (*P* < 0.001) (Fig. 6B).

## 4 Discussion

To establish and survive within mammalian cells, intracellular parasites need to modulate the host cell. In the case of *T. annulata* and *T. parva*, this includes preventing the host cell from entering apoptosis pathways and inducing their uncontrolled cellular division. The mechanisms involved have been extensively studied, but have not been fully elucidated (reviewed by Dobbelaere and Kuenzi, 2004; Heussler et al., 2006; Plattner and Soldati-Favre, 2008). The transformation of bovine mϕ by *T. annulata* infection is associated with loss of characteristic mϕ functions and phenotypic markers, which has led to the hypothesis that the host cell has de-differentiated (Sager et al., 1997). A previous investigation of early events during *T. annulata* infection revealed that the transcription factor c-MAF, a known marker of mϕ differentiation (Martinez et al., 2006; Liu et al., 2008) and an oncogene (Kataoka et al., 1993), was modulated and, therefore, may play a role in the transformation event (Jensen et al., 2008). We believe our further studies, reported here, have provided the first direct evidence that *T. annulata* modulates the mϕ differentiation state, by suppressing c-MAF at the RNA level. Furthermore, RUNX1, which is also a marker of bovine monocyte to mϕ differentiation, was suppressed in *T. annulata*-infected cells together with Pu.1 and MAFB, other transcription factors involved in mϕ differentiation.

The progression of monocyte and mϕ differentiation is orchestrated by the coordinated activity of transcription factors, including Pu.1, RUNX1, CCAAT/enhancer binding proteins (CEBPs), Sp1, MAFB and c-MAF. The expression of CEBPs was not investigated due to difficulties designing suitable primers. RUNX1 principally plays an essential role in regulating very early events in haematopoiesis (Friedman, 2002, 2007; Rosenbauer and Tenen, 2007). However, conditional ablation of RUNX1 was associated with mild myeloproliferation, including increased numbers of myeloid progenitor cells (Growney et al., 2005), supporting a role for RUNX1 in later stages of the myeloid differentiation pathway. In contrast, Pu.1 is expressed throughout the differentiation programme, from early progenitor cell to mϕ (Rosenbauer and Tenen, 2007). Both c-MAF and MAFB are important in the development of mϕ, with the up-regulation of both inducing monocyte and mϕ differentiation (Hegde et al., 1999; Kelly et al., 2000). Both MAF transcription factors act partly by repressing the expression of non-monocyte/mϕ genes (Sieweke et al., 1996; Hegde et al., 1998). The transcription factor Sp1, which plays a limited role in monocyte differentiation (Hume and Himes, 2003), was found not to be affected by *T. annulata* infection. In contrast, CSF1R was profoundly down-regulated in *T. annulata*-infected cells compared with uninfected mϕ. CSF1 is essential for mϕ survival (reviewed by Barreda et al., 2004) and it is, therefore, paradoxical that its receptor is down-regulated to such an extent in a transformed mϕ cell-line.

The level of *T. annulata*-induced suppression differed considerably amongst the transcription factors investigated, with the greatest effect on the MAF transcription factors. However, c-MAF levels only increased in four out of the five tested *T. annulata*-infected cell-lines after buparvaquone treatment, while MAFB levels increased in all of the cell-lines. This result suggests that MAFB is the more important target for suppression by *T. annulata* and indeed MAFB exhibited the greatest suppression in *T. annulata-*infected cells. However, there is functional redundancy between c-MAF and MAFB (Aziz et al., 2006), which may explain the parasite-induced suppression of c-MAF.

The variable regulation of transcription factors involved in monocyte/mϕ differentiation may account for the novel phenotype of *T. annulata*-infected cells, which are distinct from any mϕ progenitor cell. Therefore, it is more accurate to describe the effect of the parasite as ‘modulation of differentiation’ rather than de-differentiation. The balance of Pu.1 and MAFB expression has been shown to specify mϕ or DC cell fate (Bakri et al., 2005). High Pu.1 levels relative to MAFB were associated with DC differentiation from human peripheral monocytes, whilst higher MAFB levels were associated with mϕ differentiation (Bakri et al., 2005). Whilst the balance between MAFB and Pu.1 was not measured in this study, MAFB is down-regulated to a much greater extent than Pu.1 in *T. annulata*-infected cells, over 9900- and 10-fold, respectively, compared with levels in monocytes, which would alter the balance between the transcription factors and may be associated with the infected cells developing DC-like characteristics, e.g. enhanced antigen presenting capabilities and up-regulation of BoLA class II molecules (Glass and Spooner, 1990; Brown et al., 1995).

In addition to their involvement in monocyte/mϕ development, c-MAF and MAFB are involved in a range of cellular events, which may explain why *T. annulata* appears to specifically target these genes. For example, c-MAF prevents the nuclear translocation of the c-REL component of NF-κB, which potentially affects the expression of many NF-κB target genes (Homma et al., 2007). NF-κB is constitutively activated in *T. annulata*-infected cells due to the degradation of IκBs, the cytoplasmic inhibitors of NF-κB, by the IκB signalosome, which is recruited in large activated foci around the schizont surface (Heussler et al., 2002). The composition of NF-κB complexes has not been investigated in *T. annulata*-infected cells. However, T lymphocytes infected with *T. parva*, which also constitutively activate NF-κB, do express c-REL. Furthermore, c-REL-containing NF-κB complexes are translocated to the host cell nucleus (Machado et al., 2000). The prevention of nuclear translocation of NF-κB complexes containing c-REL by c-MAF would partly nullify the activity of the IκB signalosome and would suggest that the suppression of MAF transcription factors is important for *T. annulata* and *T. parva* survival within the host cell. However, it is not known at this time if MAF transcription factors are suppressed in *T. parva*-infected T and B lymphocytes.

MAF transcription factors were originally identified as oncogenes and chromosomal translocation events in multiple myelomas frequently lead to the over-expression of c-MAF and MAFB (Hurt et al., 2004; Fabris et al., 2005). Moreover, the over-expression of c-MAF induces cell transformation (Kataoka et al., 1993). Therefore, it seems counter-intuitive that the expression of c-MAF and MAFB appears to be suppressed during *T. annulata* infection, which induces transformation of the host cell. However, the effects of c-MAF and MAFB are cell-type-dependent. They act as oncogenes in chicken embryonic fibroblasts, but have a tumour suppressive effect in chicken embryonic neuroretina cells (Pouponnot et al., 2006). The over-expression of c-MAF or MAFB induces increased expression of p53 and the down-regulation of B-cell CLL/lymphoma 2, which can both induce apoptosis (Hegde et al., 1999; Hale et al., 2000). Recent work has illustrated the importance of c-MAF and MAFB in regulating mϕ proliferation. Terminally differentiated mϕ from MAFB and c-MAF double knock-out mice fail to withdraw from cell-cycle progression, unlike wild-type mϕ. However, the continued proliferation of these cells is dependent on CSF1 (Aziz et al., 2007), unlike *T. annulata*-infected cell-lines which lack the receptor.

The dynamics of c-MAF and MAFB suppression differ. MAFB suppression was detected in established *T. annulata* infected cell-lines but not during the first 3 days of infection. In contrast, during early infection *T. annulata* inhibited the up-regulation of c-MAF transcription induced by in vitro differentiation of monocytes to mϕ by 80%. However, the level of infectivity achieved in the experiments reported here did not exceed 40%. Other factors may have contributed to the suppression of c-MAF; for example, the *T. annulata* sporozoite preparations contained tick material. Ticks secrete a pharmacopoeia of immunosuppressive molecules in their saliva that can inhibit mϕ and other immune cells (reviewed by Brossard and Wikel, 2004). However, uninfected *H. anatolicum* supernatants did not inhibit c-MAF expression and the role of *T. annulata* in the suppression of c-MAF was confirmed by buparvaquone treatment of infected cells. Although the up-regulation of c-MAF was suppressed in bovine monocytes during early infection, the c-MAF levels were still much higher than those observed in established *T. annulata*-infected cell-lines. This may be due to only 40% of cells being infected and, therefore, the majority of monocytes differentiate as normal and produce high levels of c-MAF. The c-MAF levels during the differentiation process appear to be higher than that in mϕ, at least in the in vitro-derived mϕ generated in this study, which may be too high for the repression mechanism to overcome. Alternatively, the mechanism of repression may not be fully activated until a later stage of infection, when MAFB is suppressed. The maximal repression of CD14 and CD11b surface protein levels is only achieved 30 days after *T. annulata* infection (Sager et al., 1997).

In total seven genes previously reported to be regulated by MAF transcription factors in other species (Hegde et al., 1999; Cao et al., 2002, 2005; Hurt et al., 2004; Gilmour et al., 2007) were investigated in this study. The expression of four, ITGB7, CCR1, CSF2 and IL12A, were significantly correlated to c-MAF or MAFB expression. Surprisingly, no correlation was found between IL10 and c-MAF expression, although the transcription factor has previously been shown to be essential for IL10 expression induced by LPS stimulation of murine mϕ (Cao et al., 2005). However, IL10 is constitutively expressed in *T. annulata-*infected cell-lines (Brown et al., 1995) and, therefore, must be under the control of additional transcription factors, e.g. AP-1, which are regulated by T. annulata (Baylis et al., 1995). Similarly, CD14 expression, which is induced by c-MAF (Hegde et al., 1999), was not consistently up-regulated by buparvaquone treatment, with only two out of the five cell-lines exhibiting elevated CD14 mRNA levels. However, a previous study found that less than 30% of buparvaquone-treated *T. annulata*-infected cells showed increased surface expression of CD14 after 3 days treatment (Sager et al., 1997), which is in agreement with our results, implying that other factors are dominant over c-MAF and MAFB in the regulation of CD14 transcription.

Both subunits of IL12 have previously been shown to be inhibited by c-MAF (Cao et al., 2002). However, in this study c-MAF levels only correlated with IL12A expression. The regulation of IL12B by c-MAF is associated with changes in DNA-protein complexes at the IL12B promoter, which includes proteins shown to be modulated by *T. annulata* infection, e.g. NF-κB components and Pu.1 (Cao et al., 2002), which may affect IL12B transcription. In contrast, the suppression of IL12A is predominantly due to c-MAF preventing the nuclear translocation of the c-REL (Homma et al., 2007), which is essential for IL12A expression (Grumont et al., 2001). The expression of CSF2 was found to be negatively correlated to c-MAF expression. However, c-MAF has previously been shown to up-regulate CSF2 expression in T lymphocytes (Gilmour et al., 2007). The reason for this discrepancy is unclear at this time, but may relate to the difference in cell type or the involvement of other genes modulated by *T. annulata*.

The mechanism behind *T. annulata* induced suppression of the transcription factors identified in this study is unknown at this time. The transcription factor genes may be the direct target of parasite proteins or their suppression could result from the regulation of up-stream events in the monocyte/mϕ differentiation pathway. Whilst considerable efforts have been made to understand the mechanisms behind the reversible transformation event from the perspective of the bovine cell, less effort has been made to identify the parasite proteins responsible for hijacking the bovine signalling pathways. The *T. annulata* genome has now been sequenced (Pain et al., 2005) and 244 parasite-encoded proteins have been identified as candidate effector molecules (Shiels et al., 2006). These fulfil several criteria, being: unique to *Theileria*, common to both *T. annulata* and *T. parva*, specifically expressed in schizonts and containing a putative peptide sequence for translocation across the parasite membrane into the bovine cell. Of these candidates only one family, the TashATs, have been shown to localize to the bovine nucleus and modulate gene expression (Shiels et al., 2004; Oura et al., 2006). Transfection of one family member, SuAT1, altered the morphology of the bovine mϕ cell line BoMac and modulated the expression of cytoskeletal proteins, including actin (Shiels et al., 2004). Interestingly, MAFB^−/−^ mϕ also exhibit an altered morphology, associated with the up-regulation of genes involved in actin organization (Aziz et al., 2006). However, the modulation was dissimilar to that observed in SuAT1-transfected BoMac cells; for example SuAT1 expression decreased actinin levels, whereas this was enhanced in MAFB^−/−^ mϕ (Aziz et al., 2006). However, there are 17 TashAT family members and the functions of the majority are unknown.

In summary, the data presented here support the hypothesis that *T. annulata* modulates the differentiation state of the host mϕ to a novel phenotype which is beneficial for parasite survival. In particular, *T. annulata* regulates the MAF transcription factors, which play an important role in mϕ proliferation, differentiation and survival. The multiple functions of these transcription factors may explain why the parasite specifically targets these genes. Therefore, further investigation of the MAF transcription factors may lead to novel control strategies in the future.

## Figures and Tables

**Table 1:** Details of the quantitative reverse transcription-PCR primers and amplicons.

Gene	Gene symbol	Orientation	Primer sequence (5’–3’)	Size (bp)
Chemokine (C–C motif) receptor 1	CCR1	F	AAA TGA GAA GAA GGC CAA AGC	140
		R	TGC TCT GCT CAC ACT TAC GG	
Colony stimulating factor 1 receptor	CSF1R	F	ACC TTG ACA TTG GAG CCT GA	149
		R	CGG AAG TCG GAT TGT TGA GA	
Colony stimulating factor 2	CSF2	F	CAG CCC AGA AGT GAA GCA G	116
		R	GGT CCC TCC AGT GTG AAG A	
Integrin, beta 7	ITGB7	F	AGT GCG ACG ACG GCT ACT AT	214
		R	TGG TTG TCC TGG GTT CTC TC	
IL10	IL10	F	TGG ATG ACT TTA AGG GTT AC	183
		R	AGG GCA GAA AGC GAT GAC	
IL12A (p35)	IL12A	F	CAG CAA CAC GCT ACA GAA GG	149
		R	CCA GGC AAC TCT CAT TCG	
IL12B (p40)	IL12B	F	GCC TGC TTA TTG AGG TCG TG	109
		R	AGG TTC TTG GGT GGG TCT G	
Runt-related transcription factor 1	RUNX1	F	CAG GTT TGT CGG TCG GAG T	100
		R	TTT GAT GGC TCT GTG GTA GGT	
Sp1 transcription factor	SP1	F	GGT CTT CTC CAG TTT GAT TTC C	116
		R	AGG CTG TGG TTG TGA TAA TGA G	
Spleen focus forming virus (SFFV) proviral integration oncogene spi1	Pu.1	F	CGT TCC AGT TCT CGT CCA	148
		R	TTC TTC TTG ACC TTC TTG ACC T	
v-maf Musculoaponeurotic fibrosarcoma oncogene homolog	c-maf	F	TGT TGC CGA AAG CAG TCT AA	120
		R	AGA AAC CCA TAG CAT TCC ACA	
v-maf Musculoaponeurotic fibrosarcoma oncogene homolog B	mafB	F	CCC AAC ACT GGC AAG ACA TT	86
		R	TCT CCA AAG CAG GGA AAG AA	
Zinc finger protein 828	ZNF828	F	AGC AGT GAC CAA GAG CAG GT	205
		R	TCA TAG CAC GAC AGC AAC AA	

F and R denote forward and reverse primers, respectively.

## References

[bib1] Aziz A., Vanhille L., Mohideen P., Kelly L.M., Otto C., Bakri Y., Mossadegh N., Sarrazin S., Sieweke M.H. (2006). Development of macrophages with altered actin organization in the absence of mafB. Mol. Cell. Biol..

[bib2] Aziz, A., Sarrazin, S., Sieweke, M.H., 2007. Re-activation of M-CSF dependent proliferation in terminally differentiated c-maf/mafB deficient monocytes and macrophages. Keystone Symposium: The macrophage; homeostasis, immunoregulation and disease. abstract 314.

[bib3] Bakri Y., Sarrazin S., Mayer U.P., Tillmanns S., Nerlov C., Boned A., Sieweke M.H. (2005). Balance of MafB and PU.1 specifies alternative macrophage or dendritic cell fate. Blood.

[bib4] Barreda D.R., Hanington P.C., Belosevic M. (2004). Regulation of myeloid development and function by colony stimulating factors. Dev. Comp. Immunol..

[bib5] Baumgartner M., Chaussepied M., Moreau M.F., Werling D., Davis W.C., Garcia A., Langsley G. (2000). Constitutive PI3-K activity is essential for proliferation, but not survival, of *Theileria parva*-transformed B cells. Cell. Microbiol..

[bib6] Baylis H.A., Megson A., Hall R. (1995). Infection with *Theileria annulata* induces expression of matrix metallopreoteinase and transcription factor AP-1 in bovine leucocytes. Mol. Biochem. Parasitol..

[bib7] Brooke G.P., Parsons K.R., Howard C.J. (1998). Cloning of two members of the SIRP alpha family of protein tyrosine phosphatase binding proteins in cattle that are expressed on monocytes and a subpopulation of dendritic cells and which mediate binding to CD4 T cells. Eur. J. Immunol..

[bib8] Brown C.G.D., Taylor A.E.R., Baker J.R. (1987). Theileriidae. *In Vitro* Methods of Parasite Cultivation.

[bib9] Brown D.J., Campbell J.D.M., Russell G.C., Hopkins J., Glass E.J. (1995). T cell activation by *Theileria annulata*-infected macrophages correlates with cytokine production. Clin. Exp. Immunol..

[bib10] Brossard M., Wikel S.K.W. (2004). Tick immunobiology. Parasitology.

[bib11] Campbell J.D.M., Howie S.E.M., Odling K.A., Glass E.J. (1995). *Theileria annulata* induces aberrant T cell activation *in vitro* and *in vivo*. Clin. Exp. Immunol..

[bib12] Cao S., Liu J., Chesi M., Bergsagel P.L., Ho I.C., Donnelly R.P., Ma X. (2002). Differential regulation of IL-12 and IL-10 gene expression in macrophages by the basic leucine zipper transcription factor c-maf fibrosarcoma. J. Immunol..

[bib13] Cao S., Liu J., Song L., Ma X. (2005). The protooncogene c-maf is an essential transcription factor for IL-10 gene expression in macrophages. J. Immunol..

[bib14] Chaussepied M., Lallemand D., Moreau M.F., Adamson R., Hall R., Langsley G. (1998). Upregulation of Jun and Fos family members and permanent JNK activity lead to constitutive AP-1 activation in *Theileria*-transformed leukocytes. Mol. Biochem. Parasitol..

[bib15] Chaussepied M., Michie A.M., Moreau M.F., Harnett M.M., Harnett W., Langsley G. (2006). Notch is constitutively active in *Theileria*-transformed B cells and can be further stimulated by the filarial nematode-secreted product, ES-62. Microbes Infect..

[bib16] Dessauge F., Hilaly S., Baumgartner M., Blumen B., Werling D., Langsley G. (2005). C-Myc activation by *Theileria* parasites promotes survival of infected B-lymphocytes. Oncogene.

[bib17] Dobbelaere D.A.E., Kuenzi P. (2004). The strategies of the *Theileria* parasite: a new twist in host-pathogen interactions. Curr. Opin. Immunol..

[bib18] Eichhorn M., Dobbelaere D.A.E. (1994). Induction of signal transduction pathways in lymphocytes infected with *Theileria parva*. Parasitol. Today.

[bib19] Fabris S., Agnelli L., Mattiolli M., Baldini L., Ronchetti D., Morabito F., Verdelli D., Nobili L., Intini D., Callea V., Stelitano C., Lombardi L., Neri A. (2005). Characterization of oncogene dysregulation in multiple myeloma by combined FISH and DNA microarray analyses. Genes Chromosomes Cancer.

[bib20] Forsyth L.M.G., Minns F.C., Kirvar E., Adamson R.E., Hall F.R., McOrist S., Brown C.G.D., Preston P.M. (1999). Tissue damage in cattle infected with *Theileria annulata* accompanied by metastasis of cytokine-producing, schizont-infected mononuclear phagocytes. J. Comp. Pathol..

[bib21] Friedman A.D. (2002). Transcriptional regulation of granulocyte and monocyte development. Oncogene.

[bib22] Friedman A.D. (2007). Transcriptional control of granulocyte and monocyte development. Oncogene.

[bib23] Galley Y., Hagens G., Glaser I., Davis W., Eichhorn M., Dobbelaere D. (1997). Jun NH_2_-terminal kinase is constitutively activated in T cells transformed by the intracellular parasite *Theileria parva*. Proc. Natl. Acad. Sci. USA.

[bib24] Geissmann F., Auffray C., Palframan R., Wirrig C., Ciocca A., Campisi L., Narni-Mancinelli E., Lauvau G. (2008). Blood monocytes: distinct subsets, how they relate to dendritic cells, and their possible rotes in the regulation of T-cell responses. Immunol. Cell Biol..

[bib25] Gilmour J., Cousins D.J., Richards D.F., Sattar Z., Lee T.H., Lavender P. (2007). Regulation of GM-CSF expression by the transcription factor c-maf. J. Allergy Clin. Immunol..

[bib26] Glass E.J., Jensen K. (2007). Resistance and susceptibility to a protozoan parasite of cattle – gene expression differences in macrophages from different breeds of cattle. Vet. Immunol. Immunopathol..

[bib27] Glass E.J., Spooner R.L. (1990). Parasite-accessory cell interactions in theileriosis. Antigen presentation by *Theileria annulata*-infected macrophages and production of continuously growing antigen-presenting cell lines. Eur. J. Immunol..

[bib28] Glass E.J., Innes E.A., Spooner R.L., Brown C.G.D. (1989). Infection of bovine monocyte/macrophage populations with *Theileria annulata* and *Theileria parva*. Vet. Immunol. Immunopathol..

[bib29] Glass E.J., Preston P.M., Springbett A., Craigmile S., Kirvar E., Wilkie G., Brown C.G.D. (2005). *Bos taurus* and *Bos indicus* (Sahiwal) calves respond differently to infection with *Theileria annulata* and produce markedly different levels of acute phase proteins. Int. J. Parasitol..

[bib30] Growney J.D., Shigematsu H., Li Z., Lee B.H., Adelsperger J., Rowan R., Curley D.P., Kutok J.K., Akashi K., Williams I.R., Speck N.A., Gilliland D.G. (2005). Loss of Runx1 perturbs adult hematopoiesis and is associated with a myeloproliferative phenotype. Blood.

[bib31] Grumont R., Hochrein H., O’Keeffe M., Gugasyan R., White C., Caminschi I., Cook W., Gerondakis S. (2001). C-rel regulates interleukin 12 p70 expression in CD8+ dendritic cells by specifically inducing p35 gene transcription. J. Exp. Med..

[bib32] Guergnon J., Dessauge F., Traincard F., Cayla X., Rebollo A., Bost P.E., Langsley G., Garcia A. (2006). A PKA survival pathway inhibited by DPT-PKI, a new specific cell permeable PKA inhibitor, is induced by *T. Annulata* in parasitized B-lymphocytes. Apoptosis.

[bib33] Hale T.K., Myers C., Maitra R., Kolzau T., Nishizawa M., Braithwaite A.W. (2000). Maf transcriptionally activates the mouse p53 promoter and causes a p53-dependent cell death. J. Biol. Chem..

[bib34] Hegde S.P., Kumar A., Kurschner C., Shapiro L.H. (1998). C-maf interacts with c-myb to regulate transcription of an early myeloid gene during differentiation. Mol. Cell. Biol..

[bib35] Hegde S.P., Zhao J., Ashmun R.A., Shapiro L.H. (1999). C-maf induces monocytic differentiation and apoptosis in biopotent myeloid progenitors. Blood.

[bib36] Heussler V.T., Rottenberg S., Schwab R., Kuenzi P., Fernandez P.C., McKellar S., Shiels B., Chen Z.J., Orth K., Wallach D., Dobbelaere D.A.E. (2002). Hijacking of host cell IKK signalosomes by the transforming parasite *Theileria*. Science.

[bib37] Heussler V.T., Sturm A., Langsley G. (2006). Regulation of host cell survival by intracellular *Plasmodium* and *Theileria* parasites. Parasitology.

[bib38] Homma Y., Cao S., Shi X., Ma X. (2007). The Th2 transcription factor c-maf inhibits IL-12p35 gene expression in activated macrophages by targeting NF-κB nuclear translocation. J. Interferon Cytokine Res..

[bib39] Hume D.A., Himes S.R. (2003). Transcription factors that regulate macrophage development and function. Handb. Exp. Pharmacol..

[bib40] Hurt E.M., Wiestner A., Rosenwald A., Shaffer A.L., Campo E., Grogan T., Bergsagel P.L., Kuehl W.M., Staudt L.M. (2004). Overexpression of c-maf is a frequent oncogenic event in multiple myeloma that promotes proliferation and pathological interactions with bone marrow stroma. Cancer Cell.

[bib41] Iwasaki H., Akashi K. (2007). Myeloid lineage commitment from the hematopoietic stem cell. Immunity.

[bib42] Jensen K., Talbot R., Paxton E., Waddington D., Glass E.J. (2006). Development and validation of a bovine macrophage specific cDNA microarray. BMC Genomics.

[bib43] Jensen K., Paxton E., Waddington D., Talbot R., Darghouth M., Glass E.J. (2008). Differences in the transcriptional responses induced by *Theileria annulata* infection in bovine monocytes derived from resistant and susceptible cattle breeds. Int. J. Parasitol..

[bib44] Jungi T.W., Thony M., Brcic M., Adler B., Pauli U., Peterhans E. (1996). Induction of nitric oxide synthase in bovine mononuclear phagocytes is differentiation stage-dependent. Immunobiology.

[bib45] Jura W.G.Z.O., Brown C.G.D., Kelly B. (1983). Fine structure and invasive behaviour of the early developmental stages of *Theileria annulata* in vitro. Vet. Parasital..

[bib46] Kataoka K., Nishizawa M., Kawai S. (1993). Structure-function analysis of the maf oncogene product, a member of the b-Zip family. J. Virol..

[bib47] Kelly L.M., Englmeier U., Lafon I., Sieweke M.H., Graf T. (2000). MafB is an inducer of monocytic differentiation. EMBO J..

[bib48] Lehtonen A., Ahlfors H., Veckman V., Miettinen M., Lahesmaa R., Julkunen I. (2007). Gene expression profiling during differentiation of human monocytes to macrophages or dendritic cells. J. Leukoc. Biol..

[bib49] Liu H., Shi B., Huang C.C., Eksarko P., Pope R.M. (2008). Transcriptional diversity during monocyte to macrophage differentiation. Immunol. Lett..

[bib50] McGuire K., Manuja A., Russell G.C., Springbett A., Craigmile S.C., Nichani A.K., Malhotra D.V., Glass E.J. (2004). Quantitative analysis of pro-inflammatory cytokine mRNA expression in *Theileria annulata* infected cell lines derived from resistant and susceptible cattle. Vet. Immunol. Immunopathol..

[bib51] Machado J., Fernandez P.C., Baumann I., Dobbelaere D.A.E. (2000). Characterization of NF-κB complexes in Theileria parva-transformed T cells. Microbes Infect..

[bib52] Martinez F.O., Gordon S., Locati M., Mantovani A. (2006). Transcriptional profiling of the human monocyte-to-macrophage differentiation and polarization: new molecules and patterns of gene expression. J. Immunol..

[bib53] Oura C.A.L., McKellar S., Swan D.G., Okan E., Shiels B.R. (2006). Infection of bovine cells by the protozoan parasite *Theileria annulata* modulates expression of the ISGylation system. Cell. Microbiol..

[bib54] Pain A., Renauld H., Berriman M., Murphy L., Yeats C.A., Weir W., Kerhornou A., Aslett M., Bishop R., Bouchier C., Cochet M., Coulson R.M., Cronin A., de Villiers E.P., Fraser A., Fosker N., Gardner M., Goble A., Griffiths-Jones S., Harris D.E., Katzer F., Larke N., Lord A., Maser P., McKellar S., Mooney P., Morton F., Nene V., O’Neil S., Price C., Quail M.A., Rabbinowitsch E., Rawlings N.D., Rutter S., Saunders D., Seeger K., Shah T., Squares R., Squares S., Tivey A., Walker A.R., Woodward J., Dobbelaere D.A., Langsley G., Rajandream M.A., McKeever D., Shiels B., Tait A., Barrell B., Hall N. (2005). The genome of the host-cell transforming parasite *Theileria**annulata* and a comparison with *T. Parva*. Science.

[bib55] Pfaffl M.W. (2001). A new mathematical model for relative quantification in real-time RT-PCR. Nucleic Acids Res..

[bib56] Plattner F., Soldati-Favre D. (2008). Hijacking the host cellular functions by the Apicomplexa. Annu. Rev. Microbiol..

[bib57] Pouponnot C., Sii-Felice K., Hmitou I., Rocques N., Lecoin L., Druillennec S., Felder-Schmittbuhl M.P., Eychene A. (2006). Cell context reveals a dual role for maf in oncogenesis. Oncogene.

[bib58] Rosenbauer F., Tenen D.G. (2007). Transcription factors in myeloid development: balancing differentiation with transformation. Nat. Rev. Immunol..

[bib59] Rozen S., Skaletsky H.J., Krawetz S., Misener S. (2000). Primer3 on the WWW for general users and for biologist programmers. Bioinformatics Methods and Protocols: Methods in Molecular Biology.

[bib60] Sager H., Davis W.C., Dobbelaere D.A., Jungi T.W. (1997). Macrophage-parasite relationship in theileriosis. Reversible phenotypic and functional dedifferentiation of macrophages infected with *Theileria annulata*. J. Leukoc. Biol..

[bib61] Serbina N.V., Jia T., Hohl T.M., Pamer E.G. (2008). Monocyte-mediated defense against microbial pathogens. Annu. Rev. Immunol..

[bib62] Shiels B.R., McKellar S., Katzer F., Lyons K., Kinnaird J., Ward C., Wastling J.M., Swan D. (2004). A *Theileria annulata* DNA binding protein localized to the host cell nucleus alters the phenotype of a bovine macrophage cell line. Eukaryot. Cell.

[bib63] Shiels B.R., Langsley G., Weir W., Pain A., McKellar S., Dobbelaere D. (2006). Alteration of host cell phenotype by *Theileria annulata* and *Theileria parva*: mining for manipulators in the parasite genomes. Int. J. Parasitol..

[bib64] Sieweke M.H., Tekotte H., Frampton J., Graf T. (1996). MafB is an interaction partner and repressor of Ets-1 that inhibits erythroid differentiation. Cell.

[bib65] Spooner R.L., Innes E.A., Glass E.J., Brown C.G.D. (1989). *Theileria annulata* and *T. Parva* infect and transform different bovine mononuclear cells. Immunology.

